# Exploring parents' screen-viewing behaviours and sedentary time in association with their attitudes toward their young child's screen-viewing

**DOI:** 10.1016/j.pmedr.2017.06.011

**Published:** 2017-07-05

**Authors:** Emma Solomon-Moore, Simon J. Sebire, Corrie Macdonald-Wallis, Janice L. Thompson, Deborah A. Lawlor, Russell Jago

**Affiliations:** a Centre for Exercise, Nutrition and Health Sciences, School for Policy Studies, University of Bristol, Bristol, UK; b School of Sport, Exercise and Rehabilitation Sciences, University of Birmingham, Birmingham, UK; c School for Social and Community Medicine, University of Bristol, Bristol, UK; d MRC Integrative Epidemiology Unity, University of Bristol, Bristol, UK

**Keywords:** SV, screen-viewing, TV, television, IMD, indices of multiple deprivation

## Abstract

Sedentary time and screen-viewing (SV) are associated with chronic disease risk in adults. Parent and child sedentary time and SV are associated. Parents influence children's SV through parenting styles and role modelling. Understanding whether parents' attitudes toward child SV are associated with their own SV and sedentary time will aid development of family interventions to reduce sedentary behaviours. Cross-sectional data with 809 parents from Bristol, UK were collected in 2012–2013 and analysed in 2016. Parental total sedentary time was derived from accelerometer data. Parents self-reported daily television viewing, use of computers, games consoles, and smartphone/tablets (none, 1–59 min, 1–2 h, > 2 h) and attitudes toward child SV. Adjusted linear and logistic regression models were used to examine associations, separately for weekdays and weekend days. Having negative attitudes toward child SV was associated with lower weekend sedentary time (Coeff: − 6.41 [95% CI: − 12.37 to − 0.45] min/day). Limiting behaviours and having negative attitudes toward child SV were associated with lower weekday television viewing (OR: 0.72 [0.57–0.90] and 0.57 [0.47–0.70] respectively), weekend television viewing (0.75 [0.59–0.95] and 0.61 [0.50–0.75]), and weekend computer use (0.73 [0.58–0.92] and 0.80 [0.66–0.97]). Negative attitudes were also associated with lower smartphone use on weekdays (0.70 [0.57–0.85]) and weekends (0.70 [0.58–0.86]). Parent self-efficacy for limiting child SV and setting SV rules were not associated with sedentary time or SV. Reporting negative attitudes toward child SV was associated with lower accelerometer-assessed weekend total sedentary time and self-reported SV behaviours, while limiting child SV was also associated with lower self-reported SV.

## Highlights

• Limiting child SV is associated with lower TV viewing and computer use in parents.
• Having negative attitudes toward child SV is associated with lower SV in parents.
• Negative attitudes toward child SV are associated with lower parent sedentary time.
• Self-efficacy for limiting SV is not associated with parents' SV & sedentary time.
• Setting SV rules for children is not associated with parents' SV & sedentary time.

## 1 Introduction

Sedentary behaviours are defined as any waking behaviours characterised by an energy expenditure of ≤ 1.5 METS, where sitting or lying is the dominant mode of posture (e.g., screen-viewing (SV), motorised transport, office work) (The Sedentary Behaviour and Obesity Expert Working Group, 2010, Sedentary Behaviour Research Network, 2012). National data from England in 2012 suggest that adults spend approximately 5 h daily being sedentary on both weekdays and weekend days (Health and Social Care Information Centre, 2013). Moreover, half of English adults in 2012 spent two or more hours watching television (TV) or other screens daily, and a third watched TV for over 3 h (Shiue, 2016), with TV viewing the most prevalent leisure-time activity for UK adults in 2005 (Office for National Statistics, 2006).

Sedentary time and SV (TV, computers, tablets, smartphones, video games) have been found to be associated with increased risk of obesity (Blanck et al., 2007, Heinonen et al., 2013, Hu et al., 2003, Shields and Tremblay, 2008), cardiovascular disease (Dunstan et al., 2010, Ford and Caspersen, 2012, Inoue et al., 2008, Katzmarzyk et al., 2009, Stamatakis et al., 2011, Wijndaele et al., 2011), diabetes (Hu et al., 2003), cancer (Friberg et al., 2006, Howard et al., 2008), all-cause mortality (Dunstan et al., 2010, Inoue et al., 2008, Katzmarzyk et al., 2009, Stamatakis et al., 2011), mental disorders (Shiue, 2016), and poor self-rated health (Shiue, 2016) in adults. A study of Finnish adults found that each additional self-reported daily TV hour was associated with a 1.81 ± 0.44 cm larger waist circumference in women and 2.0 ± 0.44 cm in men (reference category: < 1 h; *p* < 0.0001) (Blanck et al., 2007). However, both cross-sectional and prospective studies in children and adults show little association between objectively-assessed time spent sedentary with adiposity or adverse cardio-metabolic health (van Ekris et al., 2016, Ekelund et al., 2012, Stamatakis et al., 2012, Stamatakis et al., 2015). This lack of association suggests that reporting bias may explain some of the associations with adverse outcomes seen in studies that only use self-report. An alternative explanation may be that SV is more strongly associated with negative health, for example due to an increase in snack consumption during SV (Pearson and Biddle, 2011), with measures of SV currently relying on self-reported data because objective SV measures for use in population studies do not exist. While some sedentary activities are associated with positive educational, mental and social benefits (e.g., reading, connecting with loved ones, imaginative play) (Jacobs et al., 2008), the links with adverse health outcomes, at least from self-reported data, cannot be ignored. As such, there is a need to develop effective interventions to reduce SV and sedentary time for the whole family. While reductions in sedentary time at work are desirable, it is more likely that major reductions in sedentary behaviour will come from addressing leisure-time behaviours, such as SV, and shifts toward more active travel (The Sedentary Behaviour and Obesity Expert Working Group, 2010).

To develop effective interventions to reduce SV and sedentary time among families, we must first understand how parent and child sedentary behaviours are associated, and how parents can influence their child's behaviours. Parent TV-viewing time has been found to be strongly associated with child TV-viewing across the week (Jago et al., 2012, Jago et al., 2014a). Parents who report low restriction of sedentary activities, low self-efficacy, and permissive parenting styles have children with greater levels of SV on average (Jago et al., 2011, Smith et al., 2010). Findings from a previous study using the B-Proact1v dataset, found parental self-efficacy to limit child SV was associated with child weekday TV-viewing and mediated associations between parental control and child SV (Jago et al., 2015). Beyond these observational studies, a RCT of a school-based intervention aimed at improving 9–10 year olds' physical activity and diet, reduced child-reported SV (though not their accelerometer-assessed sedentary behaviour or any of the primary outcomes) and this effect appeared to be mediated by an effect on child-reported maternal limitation of SV (Kipping et al., 2014, Lawlor et al., 2016).

These studies demonstrate that associations exist between parent and child SV time, and that parenting styles and preference for limiting child SV are associated with child SV. However, it is yet unknown whether parents' attitudes toward their child's SV are associated with their own SV and sedentary time. For instance, if parents who report more negative attitudes toward their child's SV also report less SV and spend less time being sedentary themselves, there is potential to develop interventions to encourage parents to have negative attitudes toward their child's SV with the aim of reducing both parent and child SV and sedentary time. Therefore, it is important to understand which aspects of parents' attitudes toward child SV (e.g., self-efficacy for limiting SV, preference for limiting SV, negative attitudes toward SV, setting rules about SV) are associated to parents' own SV and sedentary behaviour.

The aim of this study was to examine whether parents' attitudes toward their young child's SV behaviour was associated with their (the parents) objectively-assessed total sedentary time and self-reported SV behaviours. Specifically, it is hypothesised that parents with a more restrictive attitude toward their young child's SV (i.e., higher preference and efficacy for limiting child SV, more rules and negative attitudes toward SV) would engage in less accelerometer-assessed sedentary time and self-reported SV themselves.

## 2 Methods

### 2.1 Study sample

**Fig. 1:**
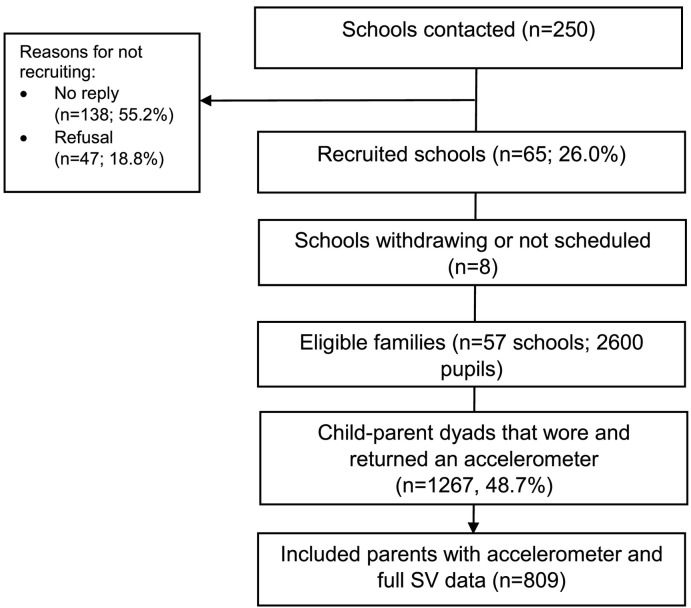
Study flow of participants.

Data are from the cross-sectional B-Proact1v study, which aimed to identify factors associated with young children's (5–6 years) and parents' physical activity and SV. Details of the study design have been reported previously (Jago et al., 2014b). Between February 2012 and May 2013, data were collected from 57 primary schools in the greater Bristol area. In total, 1267 child-parent dyads wore and returned an accelerometer and were included in the final dataset. For the current study, we were interested in parent objectively-assessed sedentary time and self-reported SV behaviours, and therefore only parents that both wore and returned an accelerometer and completed all the SV measures were included in the analyses (*n* = 809). Fig. 1 shows the study flow of participants. Ethical approval was granted by the School for Policy Studies research ethics committee at the University of Bristol, and written informed consent was obtained for all participants (Jago and Bailey, 2001).

### 2.2 Measures

#### 2.2.1 Sedentary time

Participants were asked to wear an ActiGraph GT3X waist-worn accelerometer for five consecutive days, including two weekend days, during all waking hours. Data were recorded in 10-second epochs, and uniaxial data were processed using Kinesoft (v3.3.75; Kinesoft, Saskatchewan, Canada). Accelerometer data were considered valid if participants provided at least two weekdays and one weekend day of at least 500 min of data. Three days of monitoring have previously been demonstrated to produce reliable estimates of sedentary time in adults (Dillon et al., 2016). Accelerometer “non-wear” time was defined as periods of ≥ 60 min of consecutive zero values, with an allowance of up to 2 min of interruptions, and were removed from analyses (Troiano et al., 2008). Sedentary time was determined from accelerometer data using a threshold of < 100 counts per minute (Tudor-Locke et al., 2010). Total sedentary time, including both work and leisure time, was analysed separately for weekdays and weekend days. A previous study by Clemes et al. found that objectively-assessed sedentary time was higher on workdays than non-workdays (Clemes et al., 2014).

#### 2.2.2 Self-report measures

Parents completed a questionnaire about family characteristics, personal demographics, health aspirations, home media environment, SV time, and their attitudes toward their child's SV behaviour. The Index of Multiple Deprivation (IMD) scores, based upon the English Indices of Deprivation (http://data.gov.uk/dataset/index-of-multiple-deprivation), were assigned to each family based on their reported home postcode. Home media environment was assessed by parents indicating how many of each of 10 media devices they have within the home (‘TV’, ‘DVD player’, ‘digital TV recorder’, ‘music player’, ‘desktop computer’, ‘laptop computer’, ‘tablet computer’, ‘games console’, ‘smartphone’, ‘handheld console’). The number of devices were summed to create a single score. Health aspirations were assessed on a five-item scale (‘to be physically active’; ‘to feel good about my level of physical fitness’; ‘to keep myself healthy and well’; ‘to be relatively free from sickness’; ‘to have a physically healthy lifestyle’), where parents indicated the importance of each factor using a seven-point Likert scale, from 1 ‘*not at all*’, through 4 ‘*moderately*’, to 7 ‘*very*’ (Kasser and Ryan, 1993, Kasser and Ryan, 1996). Responses were combined and the mean score used in analyses. Parent SV time was assessed via separate questions for the following SV devices: TVs, computers/laptops, games consoles, and smartphones/tablets (except for the time spent talking or texting). For each device, parents reported the time they spent using it outside of work for; a) a normal weekday, and b) a normal weekend day, with response options: ‘*none*’; ‘1–30 min’; ‘*31 min–1 h*’; ‘*1–2* *h*’; ‘*2–3* *h*’; ‘*3–4* *h*’; and ‘*4* *h or more*’. This method of self-reporting SV time has previously been used to assess SV in parents and children (Jago et al., 2011, Jago et al., 2010, Jago et al., 2008). A review found that self-reported measures of sedentary time generally showed moderate-to-high correlations for test-retest reliability and that validity correlations were higher in domain-specific measures (e.g., TV viewing, computer use) than for overall sedentary measures across an entire day (Healy et al., 2011). Weekday and weekend SV were assessed independently due to previous evidence that suggests parents report greater SV on weekends than weekdays (Sigmundova et al., 2016).

Parents' self-efficacy to limit their child's SV was assessed via three items (how much can you do to; a) ‘control the time your child spends screen-viewing’; b) ‘help your children have alternatives to screen-viewing’; c) ‘reduce the time your child spends screen-viewing’), using a five-point Likert scale ranging from 1 ‘*nothing*’ to 5 ‘*a great deal*’, adapted from Bandura's Self Efficacy Scale (Bandura, 2006). Parents' preference for limiting their child's SV time was measured via three items (I limit how long my child; a) ‘plays video games’; b) ‘can watch TV and DVDs each day’; c) ‘can use the computer for things other than homework’), using a four-point Likert scale ranging from 1 ‘*strongly disagree*’ to 4 ‘*strongly agree*’ (Davison et al., 2011). Parents' attitudes toward their child's SV were measured by asking their perspective on two statements ‘children spending several hours per day watching television or playing video games’ and ‘children spending several hours per day during leisure time using a computer or surfing the Internet’ by using four 5-point Likert scales (The Sedentary Behaviour and Obesity Expert Working Group, 2010, Sedentary Behaviour Research Network, 2012, Health and Social Care Information Centre, 2013, Shiue, 2016, Office for National Statistics, 2006) with anchor points: ‘*beneficial ≥* *harmful,*’ ‘*healthy ≥* *unhealthy,*’ ‘*useful ≥* *of no use,*’ and ‘*of no concern ≥* *of concern*’ (He et al., 2010). Parental rules governing children's SV activities were determined by asking: ‘limiting my child's amount of TV viewing, games console or computer use time is’ (response options: 1 ‘*necessary*’ to 5 ‘*unnecessary*’), and ‘I let my child decide how much TV he/she watches’ (response options: 1 ‘*never*’ to 5 ‘*always*’) (He et al., 2010). For each of the four SV exposure variables, responses to items were combined and mean scores used for analyses.

### 2.3 Statistical analysis

Distributions of exposures, outcomes and co-variables were compared between participants included in this study and those who were excluded because of key missing data (e.g., not wearing or not having sufficient valid days of accelerometer data) using means, proportions and Chi Square statistics. To explore associations between objectively-assessed total sedentary time and index of multiple deprivation, parents' health aspirations, and home media environment Spearman's correlation coefficients were used. For the associations between SV behaviours and demographic variables means and one-way ANOVA statistics were used. The vast majority of parents did not use a games console on weekdays or weekend days (> 90% and 83%, respectively), therefore this behaviour was not included in further analyses. The four exposure variables (self-efficacy for limiting child SV, preference for limiting child SV, negative attitudes toward child SV, and rules about child SV) were treated as continuous variables in all analyses. The responses to the ‘rules about child SV’ variable were flipped so that higher scores represented more restrictive parenting practices in line with the other exposure variables.

Two of the outcome variables (accelerometer-assessed total sedentary time on weekdays and weekend days) were continuous, as such multivariable linear regression models were used to examine the associations of the four exposure variables with these two sedentary time outcomes. Participant responses to the other six outcome variables (TV viewing, computer use, and smartphone/tablet use on weekdays and weekend days) were collapsed into four time categories: ‘none’, ‘1–59 min’, ‘1–2 h’ and ‘2 h or more’. As these variables were ordinal, multivariable ordered logistic regression models were used to examine the associations of the four exposure variables with each outcome variable. Ordered logistic regression assumes that the coefficients that describe the relationship between, for example, the lowest versus all higher categories of the response variable are the same as those that describe the relationship between the next lowest category and all higher categories, known as the proportional odds assumption.

To test for proportional odds, likelihood-ratio tests were conducted, and the margins command.

used. Any models that violated this assumption were analysed separately using generalised ordered logistic regression analyses (Williams, 2006).

To take account of the parents being recruited via schools, robust standard errors were used. Adjusted models were adjusted for gender, IMD score, home media environment and health aspirations, as these have previously been associated with sedentary behaviours in adults (O'Donoghue et al., 2016, Rhodes et al., 2012, Wood et al., 2015). Adjusted linear regression models with total sedentary time were also adjusted for accelerometer wear time. All analyses were performed in Stata version 14.0 (STATA, 2011).

## 3 Results

Descriptive statistics are shown in Table 1 for participants included and excluded from the dataset. Parents excluded due to missing data were more likely to be deprived, have lower health aspirations, spend less time sedentary, use computers less but smartphones/tablets more, and have lower self-efficacy for limiting child SV. Mean accelerometer wear-time for parents was 801.9 (SD = 101.0) min on weekdays and 745.2 (111.6) min on weekend days.

**Table 1:** Descriptive characteristics of the study sample (*N* = 809).

	Included (*N* = 809)	Excluded		*p*
	Mean (SD) or %	N	Mean (SD) or %	
Parent gender (% mothers)	74.4%	427	79.4%	0.05
Index of multiple deprivation^a^	13.2 (11.1)	359	18.9 (15.3)	< 0.001
Health aspirations	5.9 (1.0)	243	5.6 (1.2)	0.002
Number of media devices	10.8 (4.6)	231	10.9 (4.5)	0.85
Accelerometer-assessed total weekday sedentary time (min/day)	542.6 (91.7)	337	490.0 (101.8)	< 0.001
Accelerometer-assessed total weekend sedentary time (min/day)	497.6 (94.1)	207	453.4 (101.6)	< 0.001
Weekday television viewing		265		0.09
None	3.5%		2.3%	
1–59 min	27.7%		22.6%	
1–2 h	41.0%		40.0%	
2 + h	27.8%		35.1%	
Weekend television viewing		262		0.05
None	2.2%		3.1%	
1–59 min	13.6%		9.2%	
1–2 h	34.0%		29.0%	
2 + h	50.2%		58.8%	
Weekday leisure computer use		263		0.006
None	11.4%		19.8%	
1–59 min	48.2%		44.9%	
1–2 h	16.8%		13.3%	
2 + h	23.6%		22.1%	
Weekend leisure computer use		256		0.001
None	17.3%		27.3%	
1–59 min	51.9%		42.2%	
1–2 h	19.8%		16.0%	
2 + h	11.0%		14.5%	
Weekday smartphone use		262		< 0.001
None	36.6%		34.4%	
1–59 min	45.5%		41.6%	
1–2 h	13.5%		10.7%	
2 + h	4.5%		13.4%	
Weekend smartphone use		264		0.001
None	36.8%		33.0%	
1–59 min	42.3%		41.7%	
1–2 h	13.8%		10.2%	
2 + h	7.1%		15.2%	
Self-efficacy for limiting SV	4.6 (0.5)	258	4.5 (0.7)	0.001
Preference for limiting SV	3.4 (0.6)	251	3.3 (0.7)	0.06
Negative attitudes toward SV	3.8 (0.7)	191	3.7 (0.7)	0.15
Rules about SV	4.1 (0.8)	257	4.1 (0.9)	0.45

a Index of multiple deprivation: a higher value indicates greater deprivation.

Table 2 displays how the outcome variables vary across demographic variables. For instance, compared to mothers, fathers spent more time being sedentary, reported more computer use on weekdays, and used smartphones/tablets for longer across the week. Participants who spent more time watching TV on weekdays or using computers across the week had lower health aspirations, while participants who spent more time watching TV or using smartphones/tablets across the week had more home media devices on average.

**Table 2:** Intercorrelations, means, proportions, and ANOVA statistics for the study outcome variables and adjustment variables.

	Index of multiple deprivation^a^		Health aspirations^a^		Home media environment^a^	
	Mean (SD)	r or f (*p*)	Mean (SD)	r or F (*p*)	Mean (SD)	r or F (*p*)
Accelerometer-assessed total weekday sedentary time^b^ (min/day)		− 0.07 (0.06)		− 0.01 (0.78)		− 0.002 (0.94)
Accelerometer-assessed total weekend sedentary time (min/day)		− 0.01 (0.74)		− 0.01 (0.74)		− 0.02 (0.57)
Weekday television viewing		2.41 (0.07)		4.34 (0.005)		12.63 (< 0.001)
None	9.9 (6.4)		6.1 (0.9)		8.1 (4.7)	
1–59 min	12.0 (9.5)		6.0 (0.9)		9.7 (4.3)	
1–2 h	13.6 (12.0)		5.9 (0.9)		11.1 (4.5)	
2 + h	14.2 (11.6)		5.7 (1.1)		11.8 (4.7)	
Weekend television viewing		0.92 (0.43)		1.83 (0.14)		11.55 (< 0.001)
None	14.7 (10.3)		5.7 (1.1)		7.4 (4.3)	
1–59 min	12.7 (10.0)		6.0 (0.9)		9.5 (4.1)	
1–2 h	12.5 (11.0)		5.9 (0.9)		10.4 (4.6)	
2 + h	13.8 (11.5)		5.8 (1.0)		11.57 (4.5)	
Weekday leisure computer use		0.68 (0.56)		3.44 (0.02)		0.49 (0.69)
None	14.4 (12.3)		6.1 (0.8)		10.8 (4.6)	
1–59 min	13.1 (11.1)		5.9 (1.0)		10.6 (4.5)	
1–2 h	13.7 (10.7)		5.7 (1.0)		11.1 (4.6)	
2 + h	12.6 (10.9)		5.8 (0.9)		10.9 (4.7)	
Weekend leisure computer use		0.68 (0.57)		4.31 (0.005)		0.97 (0.41)
None	13.4 (10.9)		6.1 (0.8)		11.0 (4.5)	
1–59 min	12.9 (11.3)		5.8 (1.0)		10.8 (4.4)	
1–2 h	13.1 (10.0)		5.8 (1.0)		10.4 (4.4)	
2 + h	14.7 (12.5)		5.7 (0.9)		11.4 (5.6)	
Weekday smartphone use		0.58 (0.63)		1.79 (0.15)		19.41 (< 0.001)
None	13.3 (10.9)		5.9 (1.0)		9.3 (4.3)	
1–59 min	13.2 (11.9)		5.9 (1.0)		11.5 (4.5)	
1–2 h	12.3 (9.1)		5.7 (0.9)		12.2 (4.4)	
2 + h	15.1 (10.5)		5.7 (1.0)		12.2 (4.5)	
Weekend smartphone use		0.17 (0.91)		1.38 (0.25)		22.28 (< 0.001)
None	13.4 (11.1)		5.9 (1.0)		9.2 (4.3)	
1–59 min	12.9 (11.7)		5.9 (1.0)		11.4 (4.4)	
1–2 h	13.6 (10.6)		5.7 (1.0)		12.2 (4.7)	
2 + h	12.9 (9.2)		5.7 (0.9)		12.6 (4.1)	

a Intercorrelations presented for continuous outcome variables, and proportions in each outcome category and F-test statistics presented for categorical outcome variables.

b Mean sedentary minutes and *t*-test statistics presented for continuous outcome variables, proportions in each outcome category and χ^2^ statistics presented for categorical outcome variables.

Table 3 presents the associations between parents' attitudes toward their child's SV and their own accelerometer-assessed sedentary time. Having negative attitudes toward child SV was associated with a reduction in parents' weekend accelerometer-assessed total sedentary time, but there were no clear associations between the other three exposure variables and weekend sedentary time, and nor were there associations between any of the four exposures and weekday accelerometer-assessed total sedentary time.

**Table 3:** Linear regression analyses showing associations between parents' attitudes toward child screen-viewing and their sedentary time.

	Unadjusted		Fully adjusted^a^	
	Difference in mean sedentary time per 1 unit of each exposure [95% CI]	*p*	Difference in mean sedentary time per 1 unit of each exposure [95% CI]	*p*
Accelerometer-assessed total weekday sedentary time (min/day)^b^				
Self-efficacy for limiting SV	11.80 [− 0.01 to 23.61]	0.05	6.64 [− 1.38 to 14.66]	0.10
Preference for limiting SV	3.31 [− 7.39 to 14.01]	0.54	− 0.71 [− 8.01 to 6.60]	0.85
Negative attitudes toward SV	− 5.08 [− 14.32 to 4.15]	0.28	− 2.48 [− 8.69 to 3.72]	0.43
Rules about SV	1.16 [− 6.81 to 9.12]	0.78	− 0.33 [− 5.70 to 5.04]	0.90

Accelerometer-assessed total weekend sedentary time (min/day)				
Self-efficacy for limiting SV	5.68 [− 6.47 to 17.84]	0.36	3.86 [− 3.86 to 11.58]	0.33
Preference for limiting SV	5.48 [− 5.48 to 16.45]	0.33	− 0.41 [− 7.45 to 6.62]	0.91
Negative attitudes toward SV	− 8.50 [− 17.95 to 0.94]	0.08	− 6.41 [− 12.37 to − 0.45]	0.04
Rules about SV	− 1.43 [− 9.59 to 6.74]	0.73	− 1.48 [− 6.63 to 3.68]	0.57

All analyses take account of clustering at the school level by using robust standard errors.

a Adjusted for parent gender, index of multiple deprivation score, health aspirations, home media environment, and accelerometer wear-time on weekdays and weekend days respectively.

b The coefficients represent a per unit increase in the scores for each of the SV exposure variables. Categories for each of the SV variables were: None, 0–59 min, 1–2 h, > 2 h.

The associations between parents' attitudes toward their child's SV and their own SV behaviours are presented in Table 4. Parental self-report of having a preference for limiting child SV and having negative attitudes toward their child's SV were both associated with lower levels of reported weekday and weekend TV viewing, and lower levels of weekend computer use; negative attitudes toward child SV were also associated with lower levels of reported weekday and weekend smartphone/tablet use. Parental report of self-efficacy for limiting their child's SV and setting rules for child SV were not associated with parents' report of their own SV.

**Table 4:** Ordered logistic regression showing associations between parents' attitudes toward child screen-viewing and their SV behaviour.

	Unadjusted		Fully adjusted^a^	
	OR^b^ for an increase in the level of the SV outcome variables per 1 unit of each exposure [95% CI]	*p*	OR for an increase in the level of the SV outcome variables per 1 unit of each exposure [95% CI]	*p*
Weekday television viewing				
Self-efficacy for limiting SV	0.91 [0.71 to 1.15]	0.43	1.03 [0.81 to 1.32]	0.80
Preference for limiting SV	0.63 [0.50 to 0.79]	< 0.001	0.72 [0.57 to 0.90]	0.005
Negative attitudes toward SV	0.54 [0.45 to 0.66]	< 0.001	0.57 [0.47 to 0.70]	< 0.001
Rules about SV	0.85 [0.72 to 1.01]	0.06	0.93 [0.78 to 1.10]	0.39

Weekend television viewing				
Self-efficacy for limiting SV	0.95 [0.74 to 1.22]	0.68	1.03 [0.80 to 1.34]	0.80
Preference for limiting SV	0.67 [0.53 to 0.84]	0.001	0.75 [0.59 to 0.95]	0.02
Negative attitudes toward SV	0.58 [0.48 to 0.71]	< 0.001	0.61 [0.50 to 0.75]	< 0.001
Rules about SV	0.84 [0.71 to 0.99]	0.05	0.91 [0.76 to 1.09]	0.31

Weekday leisure computer use				
Self-efficacy for limiting SV	0.95 [0.74 to 1.21]	0.67	1.02 [0.79 to 1.31]	0.89
Preference for limiting SV	0.84 [0.68 to 1.05]	0.12	0.90 [0.72 to 1.13]	0.37
Negative attitudes toward SV	0.83 [0.69 to 1.00]	0.05	0.87 [0.72 to 1.05]	0.15
Rules about SV	0.95 [0.81 to 1.12]	0.56	0.98 [0.83 to 1.16]	0.84

Weekend leisure computer use				
Self-efficacy for limiting SV	0.78 [0.61 to 1.00]	0.05	0.87 [0.67 to 1.11]	0.26
Preference for limiting SV	0.68 [0.55 to 0.86]	0.001	0.73 [0.58 to 0.92]	0.009
Negative attitudes toward SV	0.75 [0.62 to 0.91]	0.003	0.80 [0.66 to 0.97]	0.02
Rules about SV	0.87 [0.73 to 1.02]	0.09	0.88 [0.75 to 1.05]	0.16

Weekday smartphone/tablet use				
Self-efficacy for limiting SV	1.05 [0.81 to 1.34]	0.73	1.14 [0.88 to 1.48]	0.33
Preference for limiting SV	0.77 [0.62 to 0.96]	0.02	0.89 [0.70 to 1.12]	0.31
Negative attitudes toward SV	0.67 [0.55 to 0.81]	< 0.001	0.70 [0.57 to 0.85]	< 0.001
Rules about SV	0.98 [0.83 to 1.15]	0.77	1.09 [0.92 to 1.29]	0.31

Weekend smartphone/tablet use				
Self-efficacy for limiting SV	1.03 [0.80 to 1.32]	0.82	1.12 [0.86 to 1.45]	0.40
Preference for limiting SV	0.80 [0.64 to 0.99]	0.05	0.93 [0.74 to 1.17]	0.53
Negative attitudes toward SV	0.67 [0.55 to 0.81]	< 0.001	0.70 [0.58 to 0.86]	0.001
Rules about SV	0.97 [0.82 to 1.14]	0.69	1.09 [0.92 to 1.29]	0.32

All analyses take account of clustering at the school level by using robust standard errors.

a Adjusted for parent gender, index of multiple deprivation score, health aspirations and home media environment.

b The odds ratios represent the multiplicative change in the odds of belonging to a higher category of SV associated with a unit increase in each of the attitudes to SV variables. Categories for each of the SV variables were: None, 0–59 min, 1–2 h, > 2 h.

### 3.1 Testing of proportional odds assumption

Five models violated the proportional odds assumption (weekend television viewing with negative attitudes toward SV; weekday smartphone use with preference for limiting SV; weekday smartphone use with negative attitudes toward SV; weekend smartphone use with self-efficacy for limiting SV; weekend smartphone use with negative attitudes toward SV), and thus the generalised ordered logistic regression results are presented in Table 5. For the majority of category-specific odds ratios, the associations were in the same direction as the main analysis. For parental report of negative attitudes toward child SV no associations were present with reported weekend television viewing for more than 1 h compared with less than 1 h, and with using smartphones for more than 1 min during the week and weekend compared with no use. There was an inverse association between reported preference for limiting child SV and using a smartphone/tablet for more than 1 h on a weekday, compared to less than 1 h. Similarly, self-efficacy for limiting SV was inversely associated with using a smartphone/tablet for more than 2 h on a weekend day, compared to using a smartphone for less than 2 h.

**Table 5:** Generalised ordered logistic regression analyses for the variables that violated the proportional odds assumption.

	Unadjusted		Fully adjusted^a^	
	OR for each level of the SV outcome variables per 1 unit of the exposure variable [95% CI]	*p*	OR for each level of the SV outcome variables per 1 unit the exposure variable [95% CI]	*p*
Weekend television viewing & negative attitudes toward SV				
≥ 1 min vs. none	0.26 [0.11 to 0.58]	0.001	0.27 [0.12 to 0.61]	0.002
≥ 1 h vs. < 1 h	0.75 [0.56 to 0.99]	0.04	0.79 [0.59 to 1.05]	0.11
> 2 h vs. ≤ 2 h	0.54 [0.44 to 0.67]	< 0.001	0.57 [0.46 to 0.71]	< 0.001

Weekday smartphone use & preference for limiting SV^b^				
≥ 1 min vs. none			1.01 [0.78 to 1.30]	0.95
≥ 1 h vs. < 1 h			0.71 [0.52 to 0.97]	0.03
> 2 h vs. ≤ 2 h			0.84 [0.47 to 1.51]	0.56

Weekday smartphone use & negative attitudes toward SV				
≥ 1 min vs. none	0.78 [0.63 to 0.96]	0.02	0.83 [0.67 to 1.04]	0.10
≥ 1 h vs. < 1 h	0.56 [0.43 to 0.72]	< 0.001	0.58 [0.44 to 0.76]	< 0.001
> 2 h vs. ≤ 2 h	0.32 [0.20 to 0.52]	< 0.001	0.33 [0.20 to 0.54]	< 0.001

Weekend smartphone use & self-efficacy for limiting SV				
≥ 1 min vs. none	1.14 [0.87 to 1.48]	0.34	1.25 [0.94 to 1.66]	0.13
≥ 1 h vs. < 1 h	0.92 [0.67 to 1.27]	0.62	1.01 [0.72 to 1.43]	0.94
> 2 h vs. ≤ 2 h	0.62 [0.43 to 0.89]	0.01	0.67 [0.45 to 0.99]	0.04

Weekend smartphone use & negative attitudes toward SV				
≥ 1 min vs. None	0.82 [0.66 to 1.01]	0.06	0.88 [0.71 to 1.10]	0.27
≥ 1 h vs. < 1 h	0.55 [0.42 to 0.70]	< 0.001	0.57 [0.44 to 0.74]	< 0.001
> 2 h vs. ≤ 2 h	0.35 [0.24 to 0.51]	< 0.001	0.36 [0.25 to 0.53]	< 0.001

a Adjusted for parent gender, index of multiple deprivation score, health aspirations, and home media environment.

b Unadjusted analyses for weekday smartphone use and preference for limiting SV did not violate the proportional odds assumption.

## 4 Discussion

Parents who reported more negative attitudes toward their child's SV spent less time being sedentary on weekend days, but not on weekdays. One potential explanation for the null finding on weekdays is that parents with high sedentary time may be engaged in sedentary work, which could be indicative of higher levels of education, and thus confound the association between their attitudes toward child SV and their own weekday sedentary time. As sedentary time was measured via accelerometers, and parents were not asked to report their work hours or occupation, it is not possible to know what activities parents engaged in while being sedentary.

Parents, who had greater preferences for limiting child SV and more negative attitudes toward it, reportedly watched less TV throughout the week and used computers less on weekends. Additionally, parents with more negative attitudes toward child SV used their smartphone/tablet for less time across the week. The null finding for weekday computer use may be explained by the growing popularity of portable SV devices (tablets/smartphones) and thus computers may be more commonly used for more necessary tasks that may be less influenced by attitude beliefs (Ofcom, 2015).

This is the first study to compare parents' attitudes toward child SV with parents' own sedentary time and SV behaviour, and so it was important to understand whether parents were adopting a ‘Do as I say, not as I do’ approach to parenting, or whether they also practice what they preach. Previous studies found that parents who place greater limitations on child SV also reported lower levels of child SV (Jago et al., 2011), therefore, it seems logical that similar associations would exist with parent SV behaviour, given that parent and child SV are associated (Jago et al., 2012, Jago et al., 2014a). It may be that permissive parents do not limit their child's SV behaviour because they are unwilling to cut down their own SV or sedentary time, or because they are not concerned about SV, while more authoritative parents may engage in less SV and sedentary time themselves in order to role-model ‘healthy behaviours’ for their child, or because they have negative attitudes toward SV in regards to their own health, and thus have similar attitudes toward SV for their child.

This study found no association between parents' self-efficacy to limit their child's SV or setting SV rules with either parents' own sedentary time or self-reported SV. It is plausible that some parents felt confident limiting child SV, while not even considering their own SV or sedentary time to be an issue (cognitive dissonance) (Festinger and Carlsmith, 1959). It is recommended that future studies explore this association further to examine whether parents' confidence for limiting child SV is associated with their concern and/or awareness of their own behaviours.

Five of the models assessing parents' attitudes with self-reported SV violated the proportional odds assumption, therefore, generalised ordered logistic regression analyses were conducted to provide a more comprehensive model of how associations differed across levels of SV (Williams, 2016). For instance, parents' preference for limiting child SV was associated with lower weekday smartphone use for parents who used their smartphone for at least an hour per day (compared to less than an hour), a finding that was not present in the ordered logistic models. Similarly, parents' self-efficacy for limiting child SV was associated with lower reported weekend smartphone use for parents who used their smartphone for more than 2 h per day (compared to less than 2 h). These findings demonstrate that associations between these self-reported variables are complicated, and that more advanced models, such as generalised ordered logistic regression models, are necessary to tease out the differences across outcome levels.

The Family Ecological Model illustrates the processes by which parents influence children's diet, activity, and SV behaviours (Davison and Campbell, 2005), however other studies have shown that reciprocal reinforcing relationships exist among family members, and children can influence the health behaviours of their parents (Crockett et al., 1988, Nader et al., 1989, Perry et al., 1987, Perry et al., 1988). As such, the family can be a mutually reinforcing environment in which healthy behaviours can be introduced, accepted, and maintained (Gruber and Haldeman, 2009, Wrotniak et al., 2004). Therefore, more family models are needed that account for the complexities of the reciprocal relationship between parent and child health behaviours.

The findings in this study suggest that interventions to educate parents on the ill-effects of SV in order to instil negative attitudes toward child SV and limits for such behaviours could be a potential strategy to reduce both child and parent SV and sedentary time. Indeed, interventions to reduce sedentary behaviours in young people are more likely to be effective if they involve a family component; (Biddle et al., 2014) therefore more family-based interventions to reduce SV and sedentary time are needed. One example of an intervention that successfully reduced sedentary behaviours was PACE +; a primary care-based goal-setting and counselling intervention for adolescents in the United States (Patrick et al., 2006). Parents were educated to encourage behaviour change attempts through active support, positive role modelling and praise. Self-reported sedentary time decreased from baseline to one-year follow-up to a greater extent in intervention participants versus control (− 77.7 min/day; 95% CI: − 105.8 to − 49.5) (Patrick et al., 2006). Therefore, a key target for future research would be to conduct similar interventions encouraging parents to have more negative attitudes toward their child's SV, to limit such behaviours, and be positive SV role models for their child.

## 5 Strengths and limitations

The strengths of this study are the availability of data from a reasonably-sized sample of parents, including both mothers and fathers, and that we collected data on both self-reported SV and objectively-assessed sedentary time across both weekdays and weekend days. This, in combination with questionnaire data on family demographics, parenting styles and attitudes toward child SV allows the dataset to make a novel contribution to the literature. Limitations of the study include its cross-sectional nature so causality could not be examined. ActiGraph accelerometers are waist-worn, thus are unable to distinguish between sitting and standing still, therefore devices that utilise a thigh placement would be more accurate at recording key markers of sedentary behaviour (e.g., sitting or lying posture). 458 participants were excluded from the study due to missing data (*N* = 458), which may have resulted in sampling bias, because these participants differed from included participants in terms of their time spent sedentary, use of screen devices and self-efficacy. The SV measures were self-reported, because there are no objective measures of SV available for use in large cohort studies, however this does means that reporting bias may explain some of the study findings, where parents who reported more negative attitudes toward their child's SV may have also felt obliged to report less SV behaviour for themselves (irrespective of their actual behaviour) (Matthews et al., 2012). Additionally, the ordinal nature of the SV behaviour questionnaire enabled participants to report behaviours easily, however this also necessitated the use of more complex statistical analyses with less interpretable coefficients than a standard linear or logistic regression model. It also meant that it was not possible to calculate a combined SV score.

## 6 Conclusions

Parental report of placing limitations on child SV and having negative attitudes toward it were associated with lower levels of reported TV viewing and weekend computer use among parents. Having negative attitudes toward child SV were also associated with lower levels of smartphone/tablet use and weekend total sedentary time among parents. However, parents' self-efficacy for limiting child SV and setting SV rules were not associated with either self-reported SV or accelerometer-assessed sedentary time.

## Conflicts of interest

None.

## Transparency Document

Transparency document. Image 1
